# RNA-Seq Profiling of Spinal Cord Motor Neurons from a Presymptomatic SOD1 ALS Mouse

**DOI:** 10.1371/journal.pone.0053575

**Published:** 2013-01-03

**Authors:** Urmi Bandyopadhyay, Justin Cotney, Maria Nagy, Sunghee Oh, Jing Leng, Milind Mahajan, Shrikant Mane, Wayne A. Fenton, James P. Noonan, Arthur L. Horwich

**Affiliations:** 1 Department of Genetics, Yale University School of Medicine, New Haven, Connecticut, United States of America; 2 Howard Hughes Medical Institute, Yale University School of Medicine, New Haven, Connecticut, United States of America; 3 Kavli Institute for Neuroscience, Yale University School of Medicine, New Haven, Connecticut, United States of America; 4 Program in Computational Biology and Bioinformatics, Yale University, New Haven, Connecticut, United States of America; National Institute of Health, United States of America

## Abstract

Mechanisms involved with degeneration of motor neurons in amyotrophic lateral sclerosis (ALS; Lou Gehrig's Disease) are poorly understood, but genetically inherited forms, comprising ∼10% of the cases, are potentially informative. Recent observations that several inherited forms of ALS involve the RNA binding proteins TDP43 and FUS raise the question as to whether RNA metabolism is generally disturbed in ALS. Here we conduct whole transcriptome profiling of motor neurons from a mouse strain, transgenic for a mutant human SOD1 (G85R SOD1-YFP), that develops symptoms of ALS and paralyzes at 5–6 months of age. Motor neuron cell bodies were laser microdissected from spinal cords at 3 months of age, a time when animals were presymptomatic but showed aggregation of the mutant protein in many lower motor neuron cell bodies and manifested extensive neuromuscular junction morphologic disturbance in their lower extremities. We observed only a small number of transcripts with altered expression levels or splicing in the G85R transgenic compared to age-matched animals of a wild-type SOD1 transgenic strain. Our results indicate that a major disturbance of polyadenylated RNA metabolism does not occur in motor neurons of mutant SOD1 mice, suggesting that the toxicity of the mutant protein lies at the level of translational or post-translational effects.

## Introduction

The mechanisms involved with degeneration of motor neurons in amyotrophic lateral sclerosis (ALS, Lou Gehrig's Disease) are poorly understood. Inherited forms, accounting for ∼10% of cases, are potentially informative, however. They include mutations in SOD1 (superoxide dismutase 1) [1], [2], ubiquilin [3], VCP (valosin-containing protein; p97) [4], optineurin [5], TDP43 (TAR DNA binding protein 43) [6], [7], and FUS (fused in sarcoma) [8], [9], as well as a recently identified heritable hexanucleotide repeat expansion within an intron of a gene on chromosome 9 [10], [11]. It appears that protein misfolding is involved in the gain of function associated with mutant SOD1-linked disease [2], [12], [13] and that, correspondingly, protein quality control may be affected by mutations in ubiquilin, a protein lying in the pathway of proteasomal degradation [3], [14], in VCP, a AAA+ hexameric ring assembly that acts on ubiquitinated proteins [15], [16], or in optineurin, which encodes an autophagy receptor protein [17]. Although TDP43 and FUS, like SOD1, ubiquilin, and optineurin, are found in aggregates in the cytosol of motor neurons of patients with mutations in these genes, they are RNA binding proteins in which the mutations may lead not only to their misfolding and mislocalization but also to perturbed RNA metabolism, as documented by recent studies [18], [19]. This raises the question of whether RNA metabolism is disturbed in motor neurons as a more general manifestation of disease in other forms of ALS, e.g. in mutant SOD1-associated disease. Here, we have addressed this issue by profiling RNA from laser captured spinal cord motor neurons of an SOD1 mutant transgenic mouse strain using RNA-seq, comparing it with a corresponding wild-type SOD1 transgenic. The SOD1 mutant mice were analyzed at a presymptomatic time point when significant pathologic changes were already occurring, including SOD1 aggregation in many lower motor neuron cell bodies, astrogliosis in the vicinity of such neurons, and morphologic changes in neuromuscular junctions (NMJs) in gastrocnemius muscle. We observe minimal effects on mRNA in the mutant SOD1 transgenic mice, with altered levels of only a small number of transcripts and only rare splicing differences, relative to wild-type SOD1 transgenic mice. Thus, in contrast to the substantial changes in RNA levels and splicing reported for TDP43 and FUS mutant conditions, SOD1-linked disease is not associated with major mRNA disturbance as part of the pathogenic mechanism.

## Results

### Laser capture microdissection of motor neurons

**Figure 1:**
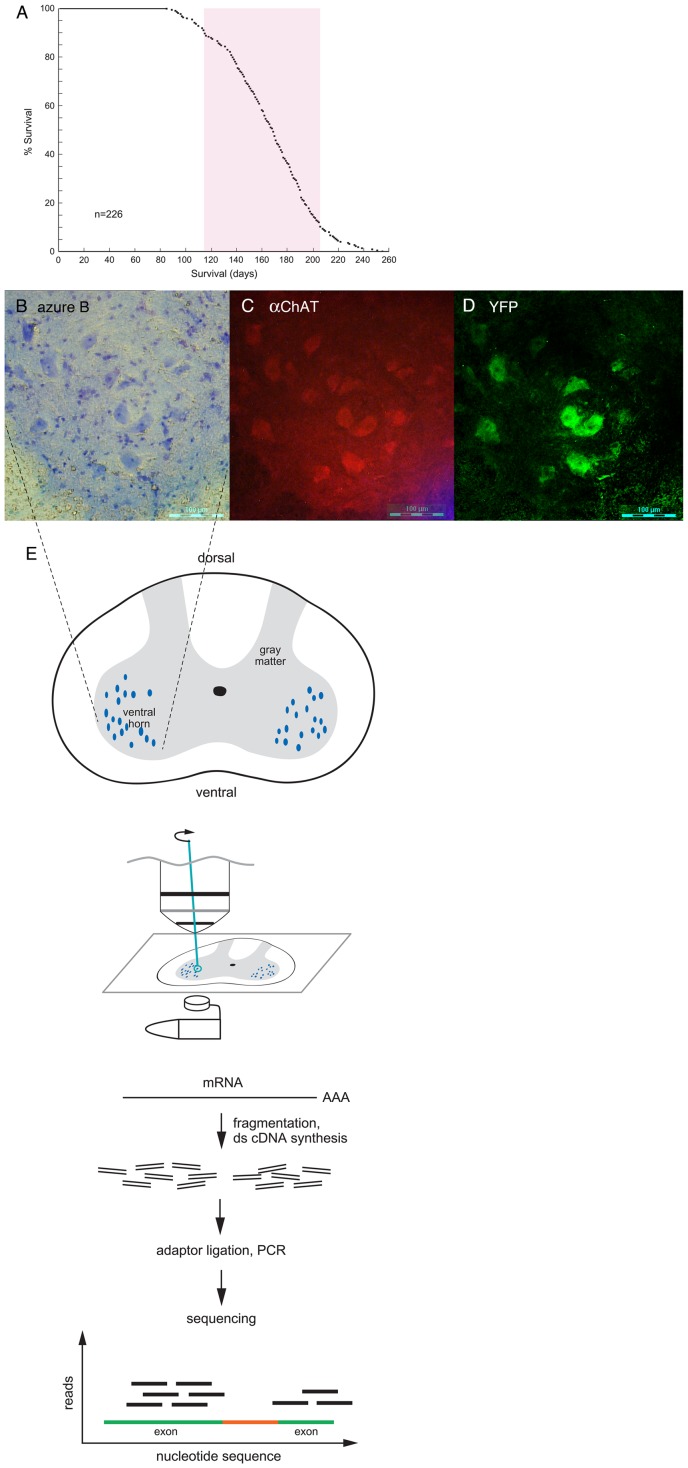
RNA-Seq of laser capture microdissected spinal cord motor neurons from wtSOD1-YFP and G85R SOD1-YFP transgenic mice. A. Survival curve of G85R SOD1-YFP mice with copy number greater than 200. 80% of the mice were paralyzed (and euthanized) between ∼115 days and 205 days (red bar). N = 226. For the present study of motor neuron RNA, presymptomatic mice at ∼90 days of age were used. B.-D. Spinal cord from a 3 month old G85R SOD1-YFP mouse. Frozen section of right ventral horn region is shown, stained with Azure B dye, panel B (see Methods); incubated with anti-ChAT antibodies, panel C; or directly examined for YFP fluorescence, panel D. The large blue-stained cell bodies in panel B are motor neurons as indicated by anti-ChAT staining in panel C. Note that the same cells have YFP fluorescence in panel D. E. Large Azure B-stained cell bodies were laser captured directly into a guanidine thiocyanate solution (see Methods) and subsequent steps carried out as diagrammed (see Methods).

To measure transcriptional differences between motor neurons of mice transgenic for G85R SOD1-YFP and wild-type SOD1-YFP, we carried out laser capture microdissection of cell bodies of large ventral horn motor neurons from spinal cords, followed by RNA-seq. The G85R SOD1-YFP strain carries over 200 copies of the transgene, being homozygous for an insertion on mouse chromosome 4, and most of these animals develop lower extremity paralysis between 4 and 6.5 months of age (Fig. 1A). The wild-type SOD1-YFP strain exhibits a similar steady-state level of fusion protein in spinal cord despite a lower transgene copy number, a function of the >10-fold more rapid turnover of the G85R SOD1-YFP as compared with wild-type [20]. The wild-type transgenic animals do not develop motor disease. We studied animals at 3 months of age, at which time nearly all G85R animals are presymptomatic, albeit that most manifest yellow fluorescent cytosolic aggregates in a fraction of motor neuron cell bodies in spinal cord (Fig. S1). At this time, there is also astrogliosis surrounding these neurons and morphologic alteration of a significant fraction of NMJs in gastrocnemius muscle (unpublished).

Freshly obtained cords were rapidly frozen in OCT embedding solution and 20 μm cryosections were stained with 1% Azure B dye in 70% ethanol (Fig. 1B; see Methods), then subjected to laser capture microdissection of large cell bodies in the ventral horn (Fig. 1E). The large cell bodies were motor neuron cell bodies, verified by anti-ChAT antibody staining carried out on the same section (Fig. 1C), and exhibited diffuse YFP fluorescence from the expressed fusion protein (Fig. 1D). Some sections from G85R animals also contained cells with large, intensely fluorescent aggregates that exhibited a darker Azure B staining pattern (Fig. S1); these were specifically excluded from laser capture.

Laser-dissected neuron cell bodies were collected directly into guanidine thiocyanate (Fig. 1E) from each of two mutant and two wild-type animals (∼4000 cells/animal), and RNA was prepared from them. The quality and quantity of RNA were determined using a Pico RNA chip on an Agilent 2100 Bioanalyzer. A typical yield from 4,000 cell bodies was ∼50 ng of total RNA, with an RNA Integrity Number (RIN) ≥8.5.

To test the purity of motor neuron RNA from laser captured cell bodies, we assessed for the presence of RNA for GFAP, a protein specific to astrocytes, which surround motor neurons and are abundant in the ventral horn. We carried out qRT-PCR on RNA from laser captured neurons and on an identical amount of RNA similarly prepared from total ventral horn. The amount of GFAP RNA in the motor neuron RNA preparation was ∼8% that in the total ventral horn RNA (Fig. S2). We thus conclude that RNA from motor neuron cell bodies was minimally contaminated with RNA from neighboring astrocytes.

### RNA-seq and differential mRNA expression in motor neurons

Total RNA recovered from motor neuron cell bodies was subjected to polyA selection, fragmentation, cDNA synthesis, adaptor ligation, and library amplification according to the standard Illumina mRNA-Seq protocol (Fig. 1E and see Methods). Seventy-five bp single-end reads were obtained from an Illumina GA IIx for two animals each of the G85R and wild-type strains. The raw data have been deposited in the GEO database, accession number GSE38820. Reads were mapped and gene expression was quantified as previously described [21]. RNA-Seq generated greater than 40 million reads for each sample, with a minimum of 65% of the reads mapping to the mouse genome at a single location for each replicate. We detected expression of 17,237 genes on average from the four samples (Table S1). Overall, gene expression was highly reproducible between the sample replicates as well as between G85R and wild-type samples (Fig. S3). Using a log-linear model coupled with likelihood ratio test (log-linear LRT), we identified only 62 genes as differentially expressed between G85R and wild-type motor neurons after multiple hypothesis testing correction (BHP <0.05) [21], [22], out of a total of 352 genes meeting the less-stringent criterion of raw p-value <0.05 (Table S2).

Using the 352-gene list, we found that genes showing reduced mRNA levels in G85R were enriched for gene ontology terms related to several aspects of neuronal function. These included neuronal cytoskeletal components involved in neurite outgrowth and axon formation such as Nefl, Nefm (neurofilament light and medium chains, respectively), and Prph (peripherin) (Table S3). Also in this category were genes associated with calcium metabolism and sensing, such as the calcitonin gene-related peptide (Cgrp) precursors, Calca and Calcb, calcineurin (Ppp3ca), and hippocalcin-like 1 (Hpcal1). Genes whose expression was increased in G85R were enriched for gene ontology terms related to mitochondrial function, ion homeostasis, and cytoplasmic vesicle formation (Table S4). These included nuclear genes encoding mitochondrial proteins primarily associated with OXPHOS complexes I, IV, and V. Overall, however, there was not a general increase in RNA levels for the majority of mitochondrial proteins encoded in the nuclear genome.

**Figure 2:**
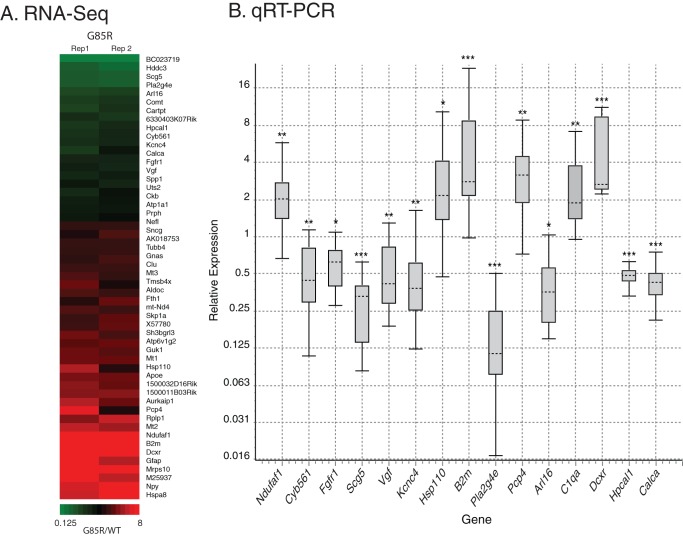
Differential expression in G85R motor neurons. A. Heatmap of selected genes from Table S2 significantly differentially expressed (raw p-value <0.005) between wild-type and G85R SOD1-YFP mice. For each gene listed, the ratio of RPKM values (G85R/WT) for individual pairs of biological replicates (Rep1, Rep2) is plotted according to the color code below. B. Validation of differentially expressed genes in G85R by qRT-PCR. Shown are box plots representing relative expression values of each gene in G85R versus wild-type motor neurons. Upper whisker represents top 25% of values, box represents the middle 50% of values, and lower whisker represents bottom 25% of values. Median value is indicated by horizontal dashed line. Statistical significance calculated by REST 2009 [37] is indicated by *  =  p<0.05, **  =  p<0.005, ***  =  p<0.0005. RNAs from at least three different mouse pairs were compared for each gene. Note that the Hsp110 and B2m validations used one exon-junction-spanning and one non-spanning primer set; minus reverse transcriptase controls for these samples were negative for DNA contamination. The expression changes in the left nine genes were validated with 0.19 ng of total RNA, while the remaining six were validated with 1.5 ng of total RNA.

To confirm the RNA-Seq results, we subjected a number of differentially expressed genes to validation by qRT-PCR. We chose genes that had at least a 1.5-fold change in expression in the mutant with a BHP value <0.05 or a raw p-value <0.05 (Fig. 2A). For each gene, multiple sets of primers were chosen that spanned exon junctions to eliminate any signal from amplification of contaminating genomic DNA; two exceptions are noted in the Fig. 2B legend. As in the RNA-Seq experiments, RNA prepared from laser captured motor neurons was employed as template for first strand cDNA synthesis. We selected 44 genes for validation by qRT-PCR using 0.19 ng of total RNA per reaction. At this level of starting material, we were only able to validate nine genes as significantly differentially expressed. However, we noted that many of the genes that failed had very high C_t_ values for both wild-type and mutant samples due to low amounts of input RNA. Therefore, a second round of validation was carried out for seven genes that had failed to validate initially, now using 1.5 ng of total RNA per reaction. This resulted in confirmed differential expression for six additional genes. In total, 15 genes were validated as significantly differentially expressed between mutant and wild-type strains by all primer sets across multiple qRT-PCR experiments (Fig. 2B). The largest changes identified by both RNA-Seq and qRT-PCR affected an Hsp110 (Hsph1) and β2 microglobulin (B2m) RNA, both >2-fold elevated, and the phospholipase A2, Pla2g4e, which was reduced by approximately 8-fold. Notably absent were changes affecting the other two Hsp110 orthologs (Hspa4 and Hspa4l) or other quality control components, e.g. from the proteasome, UPR, or mitochondrial UPR. Consistently, no significant change was detected in the RNA level of the major heat-shock transcription factor, Hsf1 (Table S1). Hsc70 (Hspa8), the major chaperone associating with G85R SOD1 in spinal cord [23], could not be evaluated because of the presence of processed pseudogenes in the mouse genome. From these data, it appears that, at this presymptomatic time when protein aggregation and gliosis, as well as effects on NMJs, are already occurring (unpublished observations), there is no global heat shock or unfolded protein response in the motor neurons. By contrast, we have validated several components involved with metabolizing or sensing calcium, such as Hpcal1, Calca, Pcp4, and Pla2g4e, suggesting a role for calcium-regulated pathways in the pathogenesis of this model system. The nature of the large diminution of Pla2g4e RNA remains unclear. Using an available antibody, we observed punctate staining for Pla2g4e in the cell bodies of wild-type motor neurons, whereas mutant cells exhibited only diffuse staining (Fig. S4). The Pla2g4e staining in wild-type cell bodies largely co-localized with the lysosomal marker Lamp2 (not shown), as would have been predicted by an earlier localization study [24].

### Lack of major disturbances of mRNA processing in the G85R mutant strain

Recent studies using a Tdp43 knockdown model for ALS have suggested that misregulation of RNA-splicing is a major contributor to ALS onset and progression in that setting [18]. In contrast, the effect here of transgenic G85R SOD1-YFP on motor neuron RNA splicing was minimal. We detected only 8 of the 287 aberrant splicing events identified in the Tdp43 study in either wild-type or G85R transcriptomes. Of these, only three genes, Eif4h, Hisppd2a, and Spp1, showed evidence of differential splicing exclusively in the G85R strain (validated by qRT-PCR; Table S5), suggesting that mutant SOD1 is associated with a different mechanism of disease causation.

In addition, RNA editing has been implicated in neuronal cell death in human ALS patients [25]. In particular, under normal conditions, nearly all GLUR2 transcripts (Gria2 in mouse) have been shown to be edited in normal motor neurons and other neuronal tissue, resulting in the change of a glutamine codon to an arginine codon. On the other hand, some spinal motor neurons of end-stage sporadic ALS patients exhibited significantly decreased editing at this position [25]. Analysis of Gria2 transcripts in the RNA-Seq data sets from our transgenic mice confirmed extensive editing of the transcript, but we detected no differences in editing efficiencies between wild-type and G85R motor neurons. Similarly, such differences in editing have not been observed in individual neurons of rats expressing H46R or G93A mutant forms of human SOD1 [26].

**Figure 3:**
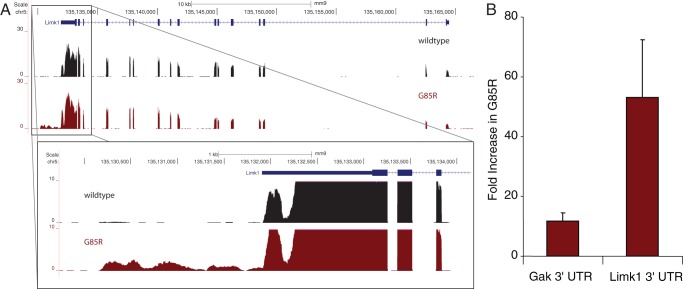
Differential expression of novel TARs in G85R motor neurons. A. RNA-Seq signal plots in reads per million mapped reads at the Limk1 gene from wild-type (black) and G85R (red) motor neurons. The canonical Limk1 gene map is shown at the top. The enlarged region shows a clear 3′-UTR extension in G85R motor neuron RNA. Similar data were observed for Gak (not shown). B. Validation of novel 3′-UTRs of Gak and Limk1 by qRT-PCR. Values are relative expression for each 3′-UTR in G85R versus wild-type motor neuron RNA.

RNA-Seq also allows the detection of regions of the genome not previously annotated as being transcribed (novel TARs). We detected 4860 novel TARs in the two strains. As was observed for mRNA expression differences, only a small fraction of these were differentially expressed in G85R, 23 reduced and 44 increased (Table S6). The collective of novel TARS likely represents a variety of RNA types, including long noncoding RNAs, enhancer RNAs, and novel UTRs of known mRNAs [27]. In order to better evaluate these sequences, we associated novel TARs upregulated in G85R with the nearest two genes. These genes were then grouped according to gene ontology using all novel TARS as a background set. We found a single gene ontology molecular function category that was enriched, that of heat shock protein binding, containing three genes (Table S7). It was not clear that the novel TAR in the neighborhood of one of these genes, Dnajb1, was actually part of that gene's transcript. In contrast, the other two genes in this category, Limk1 (Lim domain-containing kinase 1) and Gak (cyclin G-associated kinase; auxilin 2), had novel 3′-UTRs clearly associated with a fraction of the transcripts and present almost exclusively in G85R (Fig. 3A). There are no annotated ESTs or antisense transcripts in these regions, and the sequence in these locations was found to be uniquely mappable, precluding these UTRs from being overlapping transcripts or arising from artifacts in the alignment. The extensions for Limk1 and Gak are approximately 1700 nt and 1500 nt, respectively, and do not alter the coding potential of the original transcript nor create additional open reading frames. We confirmed the presence and overabundance of these novel 3′-UTRs via qRT-PCR (Fig. 3B).

## Discussion

In sum, RNA-Seq analysis of motor neurons in transgenic G85R SOD-YFP ALS animals at a presymptomatic timepoint identifies a small degree of change of only a few mRNAs, even though aggregation is already occurring in some motor neuron cell bodies, with surrounding astrogliosis, and there are morphologic NMJ abnormalities. Although it cannot be excluded that one or more of these changes, for example depression of the mRNA for Pla2g4e or other calcium-related genes identified here, could be a primary driver of mutant SOD1-linked disease, it seems more likely that the few changes at the RNA level observed here are secondary responses to the effects of high level expression of the misfolded protein in motor neurons. For example, the elevation of mRNA for the cytosolic molecular chaperone, Hsp110, is likely a stress response to the misfolded cytosolic mutant SOD1. The profile observed here contrasts with the substantial changes in mRNA biogenesis reported in the setting of TDP43 or FUS-linked pathogenesis, where both levels and splicing of many RNAs are affected [18], [19].

An earlier microarray study of RNA from laser captured motor neurons from presymptomatic G37R SOD1, as well as G85R transgenic mice likewise observed a relatively small number of genes that exhibited altered transcript levels [28]. Changes were observed in the D/L serine biosynthesis pathway, but these were specific to G37R. Changes in complement components were observed in both strains. In the present study, we detected these RNAs, but only C1qa differed significantly between G85R and wild-type animals. A microarray study of RNA from laser dissected spinal motor neurons of G93A SOD1 mice at three time points in the development of disease also found a relatively small number of changes relative to non-transgenic litter mates, particularly at later stages of disease [29]. No significant commonalities between either of the two previous studies [28], [29] and the study here were apparent, except that all found one or more components of the complement system upregulated. We validated this change in C1qa via qRT-PCR, suggesting that this difference might be involved in pathogenesis. It is not clear, however, whether this is a motor neuron derived transcript or a contaminant from inadvertently dissected surrounding activated glia, given that C1q has been implicated in neuroinflammation. Another microarray study, using laser captured motor neurons from cranial nerve nuclei and cervical spinal cord of presymptomatic G93A rats, focused on transcription differences between these regions that might account for the sparing of cranial nerves 3/4 from disease [30]. Notably, IGF-II and guanine deaminase RNAs were preferentially expressed in cranial nerve 3/4 motor neurons of G93A rats, but no comparisons were made with expression patterns in wild-type animals.

The previous laser capture studies used early versions of mouse microarrays with only single probes to several genes we have identified here, such as Pla2g4e, making them relatively insensitive to changes in such genes. In addition, these array experiments would not have been able to detect any previously unannotated transcripts such as those we observed here at the 3′-end of Limk1 and Gak, both of which could be of further interest. For example, loss of Limk1 results in the regression of presynaptic motor neuron termini in Drosophila and altered dendritic spines in mice [31], [32]. Moreover, Limk1 transcripts are translated locally in dendrites of cultured hippocampal neurons and regulated through the 3′-UTR by miR134 [33]. The extension of the 3′-UTR we have observed could introduce additional regulatory sequences affecting the maintenance of neuromuscular junctions and/or dendritic spines. The role of Gak (auxilin 2) in neurons is less well defined, but it contains an auxilin-type J-domain and interacts with Hsc70 to support clathrin uncoating and vesicle cycling in non-neuronal tissues, where it is the only auxilin [34]. It is upregulated in brains of auxilin knockout mice and supports sufficient uncoating activity to permit some animals to survive [35]; in contrast, Gak knockout is an early post-natal lethal [36].

Given the small number of changes observed here in a mutant SOD1-linked setting, it seems more likely that the primary effect(s) of the mutant SOD1 protein either lie at the level of translation or are post-translational. Post-translational effects, in particular, could involve interaction between the misfolded mutant SOD1 and cellular cytosolic or membrane proteins, which could affect their roles in macromolecular traffic, organellar function, and/or synaptic function. Further translational, morphologic, and biochemical analyses may be able to address how the mutant SOD1 protein drives motor neuron pathology.

## Materials and Methods

### Ethics statement

This study was performed in strict accordance with the recommendations in the Guide for the Care and Use of Laboratory Animals of the National Institutes of Health. All animal experiments were conducted according to a protocol approved by Yale University Institutional Animal Care and Use Committee (protocol #2011–10931).

### Animals

The transgenic mouse strains, wild-type SOD1-YFP (strain 592) and mutant G85R SOD1-YFP (strain 737), have been described [23]. The transgene copy numbers of all animals were determined by qPCR using primer sets for human genomic SOD1 and mouse genomic ApoB, as recommended by Jackson Laboratories. Animals of the 737 strain that were used for RNA isolation had apparent copy numbers between 210 and 300; copy numbers for the wild-type 592 strain were 6–8.

### RNA preparation

Wild type (strain 592) and mutant (strain 737) transgenic mice were sacrificed with a lethal dose of ketamine, followed by cardiac perfusion with PBS for 2 min. Spinal cords were dissected within 5 min, divided transversely into 9 to 10 pieces, embedded in OCT, then frozen in 2-methybutane cooled with liquid nitrogen. They were stored at −80°C until used for RNA preparation. Twenty micron frozen tissue slices, each containing 9–10 individual sections, were produced using a Leica CM3050S cryostat at −20°C and mounted on RNase-free PEN-membrane 2 μm slides (Leica); they were kept at −80°C until used. Individual slides were thawed and dried for 30–40 sec, followed by washing for 30–45 sec in RNase-free 70% ethanol for OCT removal. The following steps were then performed in order: 30–45 sec incubation in 1% Azure B (MP Biomedicals) in 70% ethanol, two 30–40 sec washes in 70% ethanol, and 40 sec air-drying. Azure B stained motor neuron cell bodies from the ventral horn from each section on the slide were laser dissected (Leica LMD6000, 20X objective) and collected in 30 μl guanidine thiocyanate buffer + DTT (RLT buffer; RNeasy Micro kit, Qiagen) into the cap of a 0.6 ml microfuge tube. Recovery of all identifiable motor neurons from the 9–10 sections on each slide (∼150 total) required <30 min. Individual collection tubes were stored at −20°C until a sufficient number had been collected. Samples were thawed and pooled, and total RNA was extracted from 3000 to 4000 motor neuron cell bodies from each animal using the RNeasy Micro kit (Qiagen) according to the protocol provided for LMD tissue. The quality of the RNA was determined on an Agilent 2100 BioAnalyzer using an RNA Pico chip. Total RNA preparations with an RNA Integrity Number (RIN) above 8.5 were used for RNA-Seq and qRT-PCR. RNA from two animals from each strain was analyzed separately by RNA-Seq, and RNA from at least 3 animals per strain was analyzed by qRT-PCR.

### Transcriptome and splicing analysis

Seventy-five bp single-end reads were mapped using Bowtie (v 0.12.3) to the mouse genome (mm9), and a custom splicing database generated from UCSC Known Gene annotation as previously described [21]–[22]. Gene expression was quantified using Cufflinks (v 0.8.3), and differential expression was determined as previously described [21]–[22].

### Quantitative reverse transcriptase PCR

Multiple sets of primers (each spanning an exon-exon junction) were designed for each mRNA of interest using Primer Blast (NCBI) against the *Mus musculus* mRNA RefSeq database (Table S8). In preliminary experiments with cDNA prepared from isolated mouse brain RNA, amplified products from each primer pair were subjected to DNA sequencing to confirm their specificity; those sets that produced incorrect or mixed sequences were not used. In general, at least two satisfactory pairs were identified for each RNA; exceptions are noted in the figure legends. cDNA from total RNA from each sample was generated using SuperScript III First Strand Synthesis System (Invitrogen) with a mixture of random hexanucleotide and oligo-dT primers. qRT-PCR reactions were performed in triplicate on a volume of cDNA corresponding to 0.19 ng or 1.5 ng of input motor neuron RNA in a 10 μL reaction volume per well using Power SYBR Green PCR Master Mix (Applied Biosystems) on an ABI PRISM 7900 (Applied Biosystems) (Fig. 2B legend). One set of mouse Gapdh primers and two sets of mouse Hprt1 primers were used as reference for all samples. The reference mRNAs showed no difference in expression between mutant and wild-type. Because of the small amount of RNA available, controls without reverse transcriptase in the first strand synthesis reaction were carried out randomly with only a few primer sets. An exception was in the study of the novel TARs for Limk1 and Gak (Fig. 3B), where the size of the extended RNA segment precluded using an exon-exon junction pair and necessitated the inclusion of this control. No amplification products were observed in any of the minus reverse transcriptase reactions, including those performed for the experiments in Fig. 3B. Fold-change between mutant and wild-type mice were determined using the ΔΔC_t_ method as implemented in the Applied Biosystems software; calculations using any of the reference sets gave the same results within experimental error.

## Supporting Information

Figure S1 **Aggregation in motor neurons of a 3 month old G85R SOD1-YFP mouse.** Image of right ventral horn of the spinal cord of a 3 month old G85R SOD1-YFP animal at the lumbar level showing: left, YFP fluorescence and, right, Azure B staining. A number of the motor neuron cell bodies have very strong local YFP fluorescence, indicative of aggregation, which has been confirmed by EM analysis (unpublished observations). The corresponding Azure B-stained cell bodies (red arrow heads) are much darker than the other cell bodies. Such motor neurons were not laser captured for this study.
(PDF)

Figure S2 **Contamination by astrocyte RNA in harvested motor neurons.** Relative expression of GFAP, an astrocyte-specific marker, in wild-type and mutant motor neurons compared to total ventral horn.
(PDF)

Figure S3 **RNA-Seq reproducibility.** Scatter plots of log_2_(RPKM) values from wild-type and G85R replicates. Pearson correlation coefficients for each comparison are indicated by R.
(PDF)

Figure S4 **Antibody staining of lumbar spinal cord section with anti-Pla2g4e antibody.** A) Representative sections are shown for 3-month old wild-type SOD1-YFP (top row) and G85R SOD1-YFP animals (bottom row); 20 µm sections from perfused animals were subjected to immunohistochemistry as described in Methods S1. Left panels, YFP fluorescence (green) with DAPI staining (blue); in the wt animal, a motor neuron in the center of the panel is strongly YFP fluorescent and, likewise, a neuron at the lower right in the mutant animal is fluorescent. Middle panels, after anti-Pla2g4e antibody staining using an Alexafluor 555 (red) secondary antibody, the wild-type motor neuron exhibits cytosolic puncta, whereas the mutant fails to show similar staining in the neuron. The wild-type did not exhibit such staining in the absence of the primary antibody (not shown). Right hand panels, merge. Magnification 100x with oil immersion objective. B) Quantification of Pla2g4e immunofluorescence. Images such as those in Fig. S4A were quantitated by counting the red Pla2g4e puncta associated with YFP-positive (green) ventral motor neurons. The percent of cells with >30 puncta (blue bars), 10–30 puncta (red bars), and <10 puncta (yellow bars) is shown for three sets of wild-type (WT) and G85R mutant animals. The number of cells evaluated for each animal is shown in parentheses. Note the large number of mutant motor neurons with <10 Pla2g4e-staining puncta compared to wild-type, where most show >30 puncta. This reduced antibody staining correlates well with the reduced mRNA levels for this protein detected by RNA-Seq (Table S2) and qRT-PCR (Fig. 2B).
(PDF)

Methods S1 (DOC)

Table S1 **Sequencing Statistics.** Total and uniquely mapped reads for wild-type and G85R motor neurons. Total genes detected as expressed for each sample are defined as any gene with an RPKM greater than zero from original read mapping. The total level of gene expression for each sample was determined as the sum of RPKM for all genes.
(XLSX)

Table S2 **Wild-type versus G85R motor neuron differential gene expression.** Log-linear analysis with LRT-statistic of wild-type and G85R motor neuron RPKMs. Columns are: UCSC cluster id, UCSC transcript id, MGI gene symbol, Entrez Gene accession number, wild-type replicate 1 RPKM, wild-type replicate 2 RPKM, G85R replicate 1 RPKM, G85R replicate 2 RPKM, log_2_ fold change, LRT statistic, raw p-value, Bonferroni corrected p-value, and Benjamani-Hochberg corrected p-value (BHP). All RPKMs were calculated by adding one read to all genes to eliminate zero values.
(XLSX)

Table S3 **DAVID output for genes with increased levels in G85R.** Full DAVID output [S2,S3] for genes identified as having increased levels in G85R from Table S2 (BHP ≤0.05 and/or raw p-value ≤0.05 and log2 fold change ≥2).
(XLSX)

Table S4 **DAVID output for genes with reduced levels in G85R.** Full DAVID output [S2,S3] for genes identified as having reduced levels in G85R from Table S2 (BHP ≤0.05 and/or raw p-value ≤0.05 and log2 fold change ≥2).
(XLSX)

Table S5 **Splice identification in wild-type and G85R motor neurons.** Identification and number of reads supporting each novel splice in each RNA-Seq replicate. Columns are: UCSC transcript id, MGI gene symbol, Entrez Gene accession number, junction coordinate, Reads mapping to junction from wild-type replicate 1, wild-type replicate 2, G85R replicate 1, and G85R replicate 2.
(XLS)

Table S6 **Differentially expressed novel TARs in G85R.** Identification and log-linear analysis with LRT-statistic of novel transcriptionally active regions in wild-type and G85R motor neurons. Columns are: novel TAR id, wild-type replicate 1 RPKM, wild-type replicate 2 RPKM, G85R replicate 1 RPKM, G85R replicate 2 RPKM, log_2_ fold change, LRT statistic, raw p-value, Bonferroni corrected p-value, and Benjamani-Hochberg corrected p-value (BHP).
(XLSX)

Table S7 **Gene ontology enrichment for genes associated by novel TARs with increased levels in G85R motor neurons.** Full GREAT output for gene ontology enrichments of genes assigned to novel TARS compared to a background set of all novel TARs identified in wild-type and G85R expression data.
(XLSX)

Table S8 **Primers used in qRT-PCR.**
(XLS)
