# Supplementary figures and images for: RNA-Seq Profiling of Spinal Cord Motor Neurons from a Presymptomatic SOD1 ALS Mouse
Source: PLoS One. 2013 Jan 3;8(1):e53575. doi: 10.1371/journal.pone.0053575 (PMC3536741; doi:10.1371/journal.pone.0053575)

YFP

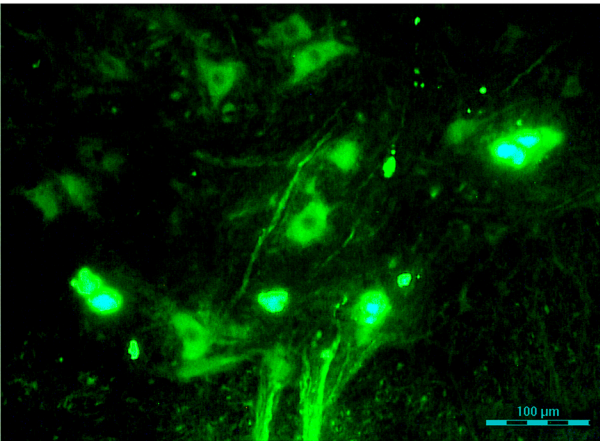

Azure B

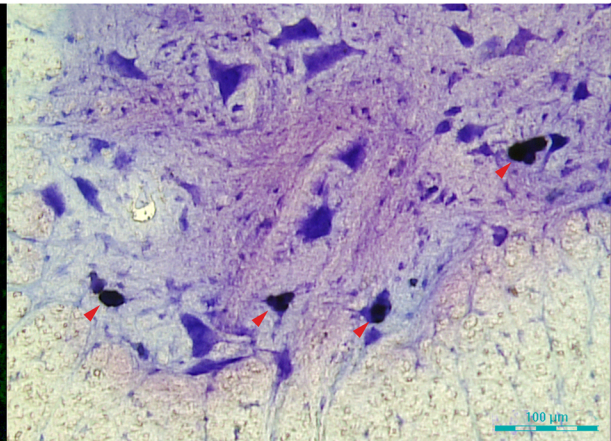

Supplement: Figure S1 — Aggregation in motor neurons of a 3 month old G85R SOD1-YFP mouse. Image of right ventral horn of the spinal cord of a 3 month old G85R SOD1-YFP animal at the lumbar level showing: left, YFP fluorescence and, right, Azure B staining. A number of the motor neuron cell bodies have very strong local YFP fluorescence, indicative of aggregation, which has been confirmed by EM analysis (unpublished observations). The corresponding Azure B-stained cell bodies (red arrow heads) are much darker than the other cell bodies. Such motor neurons were not laser captured for this study. (PDF) [file pone.0053575.s001.pdf]

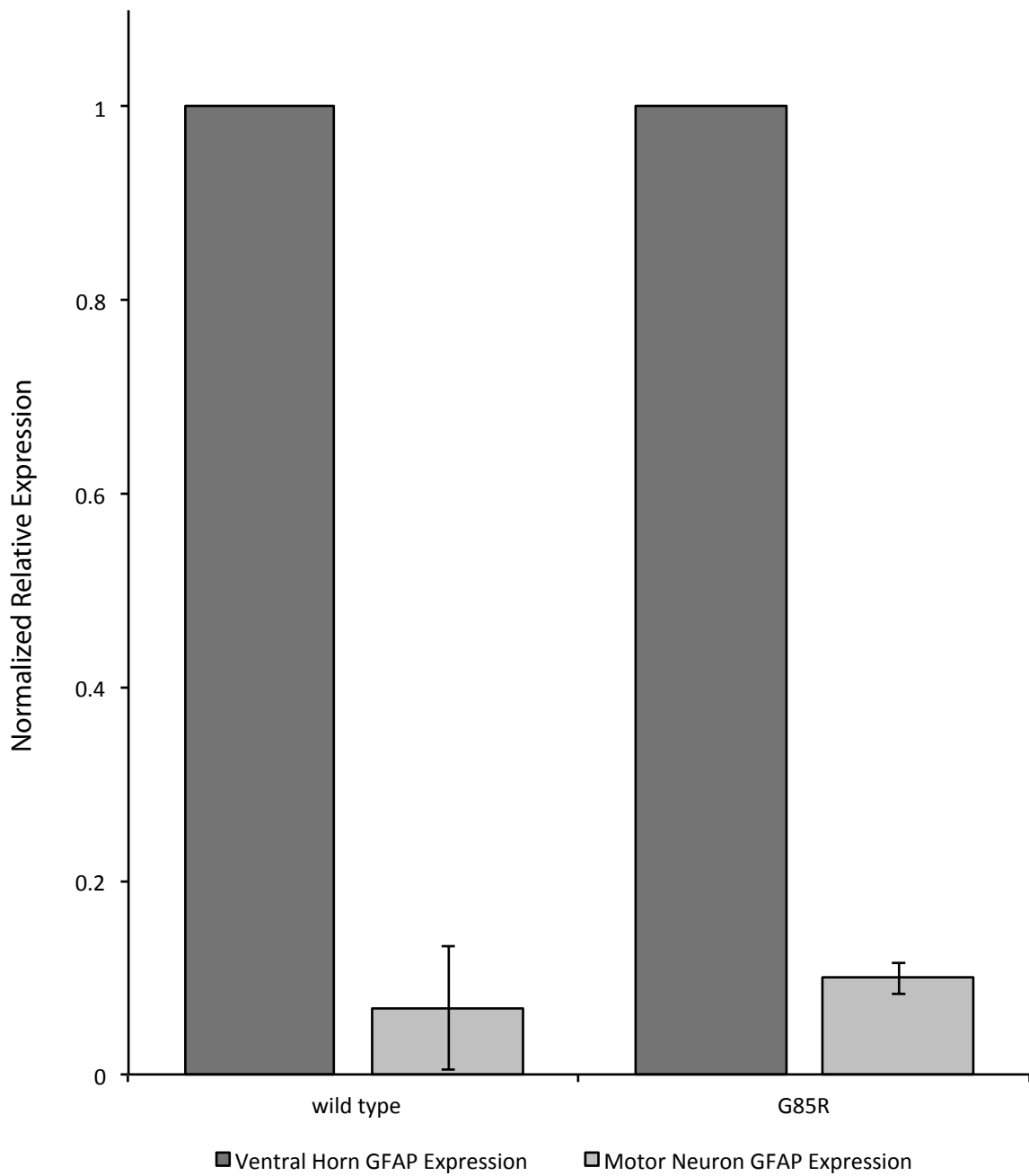

Supplement: Figure S2 — Contamination by astrocyte RNA in harvested motor neurons. Relative expression of GFAP, an astrocyte-specific marker, in wild-type and mutant motor neurons compared to total ventral horn. (PDF) [file pone.0053575.s002.pdf]

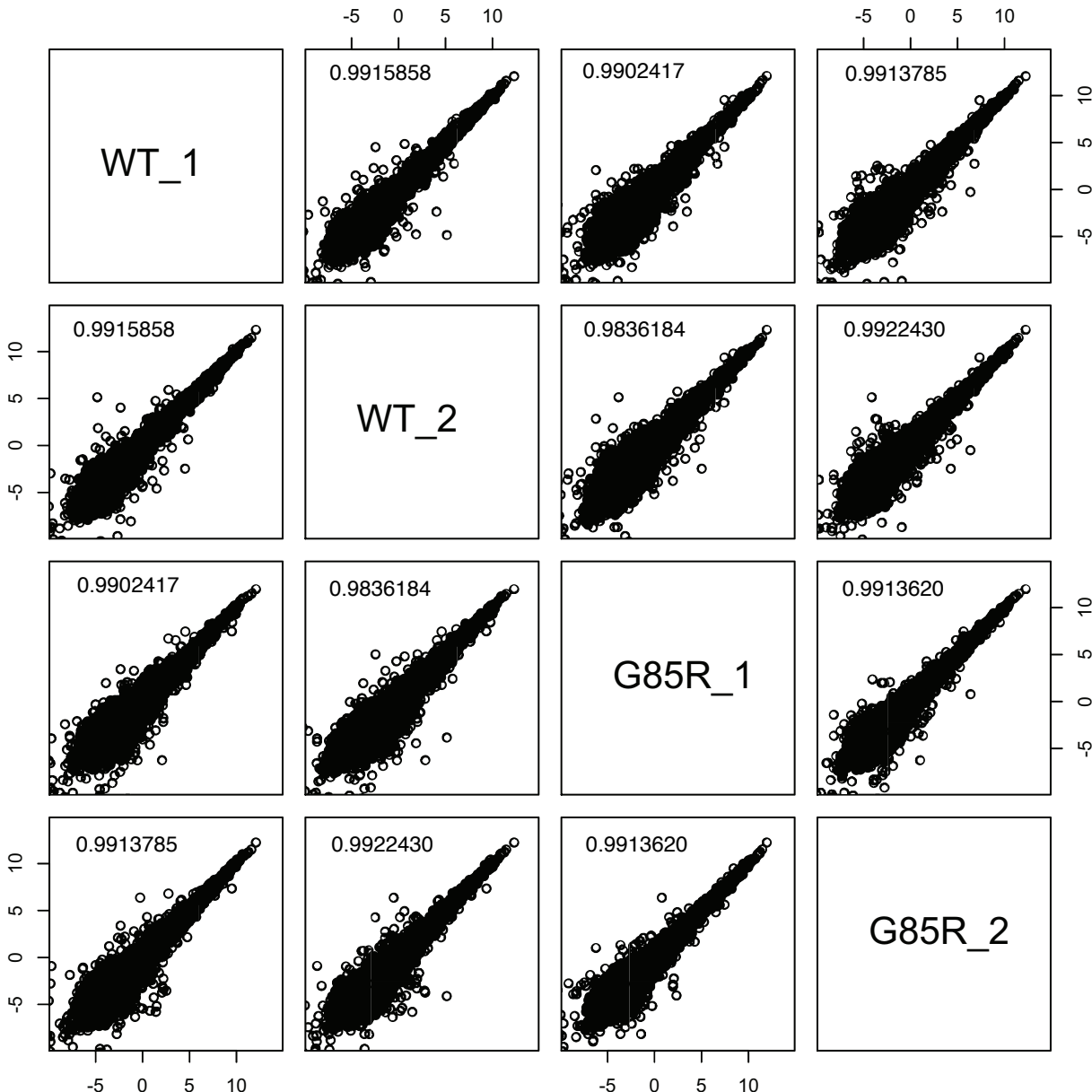

Supplement: Figure S3 — RNA-Seq reproducibility. Scatter plots of log2(RPKM) values from wild-type and G85R replicates. Pearson correlation coefficients for each comparison are indicated by R. (PDF) [file pone.0053575.s003.pdf]

A)

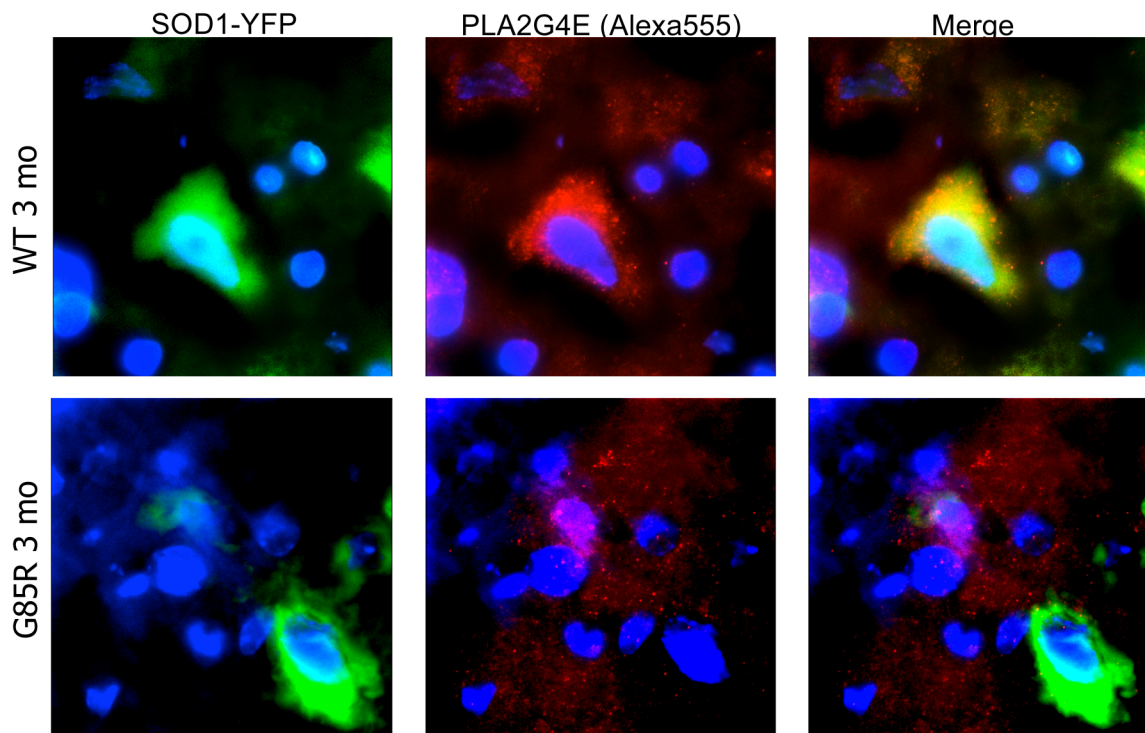

B)

## Pla2g4e IHC

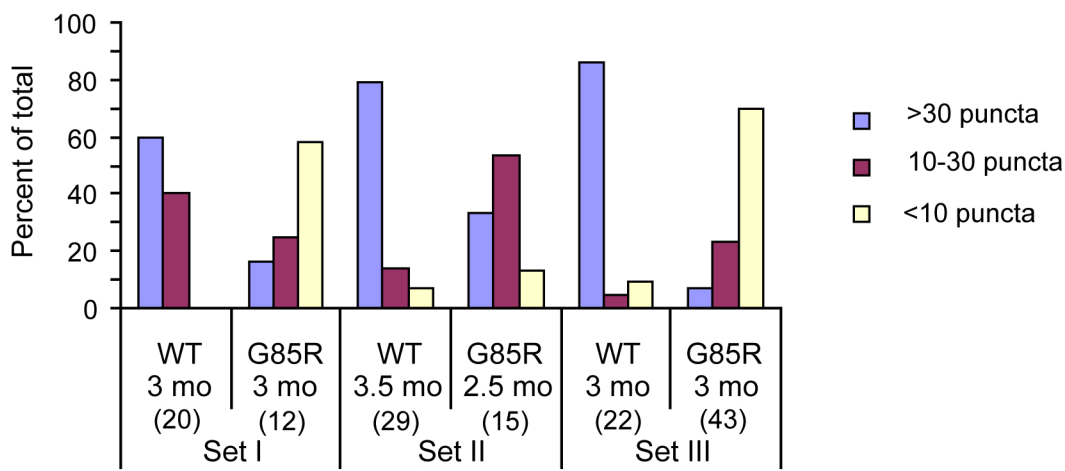

Supplement: Figure S4 — Antibody staining of lumbar spinal cord section with anti-Pla2g4e antibody. A) Representative sections are shown for 3-month old wild-type SOD1-YFP (top row) and G85R SOD1-YFP animals (bottom row); 20 µm sections from perfused animals were subjected to immunohistochemistry as described in Methods S1. Left panels, YFP fluorescence (green) with DAPI staining (blue); in the wt animal, a motor neuron in the center of the panel is strongly YFP fluorescent and, likewise, a neuron at the lower right in the mutant animal is fluorescent. Middle panels, after anti-Pla2g4e antibody staining using an Alexafluor 555 (red) secondary antibody, the wild-type motor neuron exhibits cytosolic puncta, whereas the mutant fails to show similar staining in the neuron. The wild-type did not exhibit such staining in the absence of the primary antibody (not shown). Right hand panels, merge. Magnification 100x with oil immersion objective. B) Quantification of Pla2g4e immunofluorescence. Images such as those in Fig. S4A were quantitated by counting the red Pla2g4e puncta associated with YFP-positive (green) ventral motor neurons. The percent of cells with >30 puncta (blue bars), 10–30 puncta (red bars), and <10 puncta (yellow bars) is shown for three sets of wild-type (WT) and G85R mutant animals. The number of cells evaluated for each animal is shown in parentheses. Note the large number of mutant motor neurons with <10 Pla2g4e-staining puncta compared to wild-type, where most show >30 puncta. This reduced antibody staining correlates well with the reduced mRNA levels for this protein detected by RNA-Seq (Table S2) and qRT-PCR (Fig. 2B). (PDF) [file pone.0053575.s004.pdf]
