# Supplementary material for: RNA-Seq Profiling of Spinal Cord Motor Neurons from a Presymptomatic SOD1 ALS Mouse
Source: PLoS One. 2013 Jan 3;8(1):e53575. doi: 10.1371/journal.pone.0053575 (PMC3536741; doi:10.1371/journal.pone.0053575)
Supplement: Methods S1 — (DOC) [file pone.0053575.s005.doc]

Supporting Information – Methods
Immunohistochemistry 
Immunohistochemistry was performed on 20 ìm cross-sections cut from spinal cords embedded in OCT as described in Materials and Methods, except the mouse was perfused with 2% paraformaldehyde (PFA) in PBS before removing the cord.  Tissue sections on Snowcoat X-tra slides were post-fixed using 2% PFA in PBS for 15 min.  Antigen retrieval was carried out using 0.1% trypsin + 0.1% CaCl2, pH 7.5, for 30 min at 37°C, followed by three 1-min PBS washes.  Sections were permeabilized with 0.5% Triton X-100 for 15 min at room temperature and washed as before prior to blocking with 10% donkey serum in PBS + 0.1% Triton X-100 for 30 min at 37°C.  Incubation with Pla2g4e antibody [group IVE sPLA2 (K-15); Santa Cruz Biotechnology, sc-247087] (1:100) was performed for 1 hr at 37°C followed by overnight at 4°C.  After three 3-min washes with PBS, slides were incubated with secondary antibody, Alexafluor 555 donkey á-goat IgG (Invitrogen) (1:1000), for 1 hr at room temperature in the dark. After three 3-minute washes with PBS, slides were treated with 10 mM CuSO4 in 50 mM ammonium acetate, pH 5, for 15 min at room temperature to remove lipofuscin autofluorescence [S1] prior to mounting with Vectashield containing DAPI (Vector Laboratories).  Fluorescence microscopy was performed using an Olympus IX81 microscope.
For the images in Fig. 1, a modified protocol was used.  Twenty micron sections were prepared from an OCT-embedded spinal cord that had not been perfused with PFA, mounted on Snowcoat X-tra slides, thawed, washed with cold PBS to remove the OCT, and post-fixed as above.  Sections were permeabilized with 0.5% Triton X-100 and blocked with donkey serum without an antigen retrieval step.  They were incubated with goat anti-Chat polyclonal antibody (Millipore AB144p) at 1:250 dilution overnight at 4°C.  After washing with PBS, they were incubated with Alexafluor 555 donkey anti-goat IgG (1:1000) for 1 hr at room temperature, washed with PBS, and imaged in the GFP and RFP channels on the LMD6000 microscope.  The slides were then stained with 1% Azure B in PBS for 2 min at room temperature, washed with PBS, and imaged with transmitted light on the LMD6000.

References
S1. Schnell SA, Staines WA, Wessendorf MW (1999) Reduction of lipofuscin-like autofluorescence in fluorescently labeled tissue. J Histochem Cytochem 47: 719-730.

S2. Huang DW, Sherman BT, Lempicki RA (2009) Systematic and integrative analysis of large gene lists using DAVID Bioinformatics Resources. Nature Protoc 4: 44-57.

S3. Huang DW, Sherman BT, Lempicki RA (2009) Bioinformatics enrichment tools: paths toward the comprehensive functional analysis of large gene lists. Nucleic Acids Res. 37: 1-13.

S4. McLean CY, Bristor D, Hiller M, Clarke SL, Schaar BT, et al. (2010) GREAT improves functional interpretation of cis-regulatory regions. Nat Biotechnol 28: 495-501.
